# Unravelling the DNA sequences carried by *Streptomyces coelicolor* membrane vesicles

**DOI:** 10.1038/s41598-022-21002-z

**Published:** 2022-10-05

**Authors:** Teresa Faddetta, Alberto Vassallo, Sara Del Duca, Giuseppe Gallo, Renato Fani, Anna Maria Puglia

**Affiliations:** 1 Department of Biological, Chemical and Pharmaceutical Sciences and Technology, University of Palermo, 90128 Palermo, Italy; 2 School of Biosciences and Veterinary Medicine, University of Camerino, 62032 Camerino, Italy; 3 Department of Biology, University of Florence, 50019 Sesto Fiorentino, Italy

**Keywords:** Bacterial genetics, Bacterial genomics, Bacteriology

## Abstract

Membrane vesicles (MVs) are spherical particles with nanoscale dimensions and characterized by the presence of diverse cargos, such as nucleic acids, proteins, lipids, and cellular metabolites. Many examples of (micro)organisms producing MVs are reported in literature. Among them, bacterial MVs are of particular interest because they are now considered as the fourth mechanism of horizontal gene transfer. *Streptomyces* bacteria are well-known for their ecological roles and ability to synthesize bioactive compounds, with *Streptomyces coelicolor* being the model organism. It was previously demonstrated that it can produce distinct populations of MVs characterized by different protein and metabolite cargos. In this work we demonstrated for the first time that MVs of *S. coelicolor* carry both DNA and RNA and that their DNA content represents the entire chromosome of the bacterium. These findings suggest that MV DNA could have a role in the evolution of *Streptomyces* genomes and that MVs could be exploited in new strain engineering strategies.

## Introduction

Bacterial membrane vesicles (MVs) are spherical and membranous particles with nanometer diameter and their cargo includes various macromolecules—such as nucleic acids (DNA and RNA), lipids, and proteins (i.e., enzymes and toxins)—and small molecules—such as cellular metabolites, signals, and bioactive molecules (i.e., antibiotics)^1–4^. The release of MVs can facilitate many biological functions such as nutrient uptake, competition, survival, stress response, intercellular communication, and horizontal gene transfer (HGT)^1,3,5^. In the context of HGT, which is recognized as one of the major forces driving the shaping of genes and genomes and their evolution^6^, the MVs may play a (very) important role. The main studied mechanisms of HGT in prokaryotes are transformation, conjugation, and transduction^6,7^. However, in recent years MVs have been considered as the fourth mechanism of HGT, called vesiduction^5,8–10^. Indeed, different types of genetic material, such as chromosomal, plasmid, and/or phage DNA, together with sRNA, mRNA, and miRNA, have been detected inside MVs or in association with their membrane^5,8,9,11^. The occurrence of HGT mediated by MVs has been observed in many studies: for example, Klieve et al*.*^12^ demonstrated, for the first time, the association of DNA with MVs released by Gram-positive bacteria belonging to the *Ruminococcus* genus, and the capability of these MVs to restore a specific metabolic activity in mutant strains^12^. Moreover, MVs have been reported to transfer plasmids between different strains ^13,14^ and DNA cargo into eukaryotic host cells ^15^.

In addition, extracellular DNA (eDNA) is an important component of bacterial biofilm matrix allowing cell adherence in the early stages of biofilm formation. In *Streptococcus mutans* planktonic cells eDNA is released also by a lysis-independent mode involving MVs. Thus, eDNA in MVs of planktonic cultures may play some role in the early stage of biofilm formation promoting bacterial colonization. Moreover, it is reported that MV eDNA influences biofilm structural integrity and stability^16^.

Only 4% of the studies on prokaryotic membrane vesicles conducted between 1972 and 2021 concern MVs of Gram-positive bacteria^17^: indeed, it has been assumed for a long time that Gram-positive bacteria could not produce vesicles, because of the lack of an outer membrane layer and the presence of thick cell walls^1^. In recent years, Gram-positive MVs have gained more attention and their release was demonstrated in both pathogenic and non-pathogenic strains, such as *Staphylococcus* spp., *Bacillus* spp., *Lactobacillus* spp., and *Streptomyces* spp.^4,18–21^.

*Streptomyces* is a genus of filamentous Gram-positive bacteria that are ubiquitous in terrestrial and marine environments, where they can live in symbiosis, for example, with plants, fungi, insects, and sponges^22^: adaptation to such diverse ecological niches and interaction with other (micro)organisms have led to the peculiar chemical diversity of their bioactive metabolites, whose production is regulated by a complex network of environmental signals, such as nutrient shortage and presence of competitors^22–24^. Streptomycetes have a complex life cycle as the mycelium of a colony is formed by distinct cell types (i.e., vegetative hyphae, aerial hyphae, and spores) and even different subpopulations: indeed, *Streptomyces* colonies have been recently described as similar to colonies of social insects with labor division, because of the presence of mutant cell subpopulations that are characterized by deletions and/or amplifications in the arms of their linear chromosomes, and that hyperproduce antibiotics at the expenses of their own fitness^25,26^.

In these bacteria, conjugation is the most studied mechanism of HGT, as it has been studied since the 1950s with experiments involving prototrophic strains and auxotrophic mutants^27–29^. A peculiar feature of conjugation in *Streptomyces* is the formation of the so-called pocks: when spores of a strain carrying a plasmid were co-cultivated with an excess of recipient strain spores, circular zones of growth retardation in the mycelial lawn of the recipient were observed^30^. The pocking phenotype was associated with the plasmid transfer as the donor plasmid was found in the cells forming the pock^30^. In addition, while in unicellular bacteria conjugative plasmids are transferred as a single stranded DNA from the donor to the recipient through a Type IV secretion system that involves multiple proteins, *Streptomyces* conjugation consists in the transfer of double stranded DNA and a protein encoded by the conjugative plasmid (i.e., TraB) is required^31–36^.

*Streptomyces coelicolor* is considered the model organism for the study of Streptomycetes. We have very recently demonstrated that *S*. *coelicolor* grown in liquid cultures releases MVs of different sizes and with specific protein and metabolic cargos^4^. However, it is not still clear whether specific DNA fragments or the entire set of *Streptomyces* genomic sequences can be carried by MVs. Hence the aim of this work was to perform the sequencing and analysis of DNA purified from *S. coelicolor* MVs in order to check whether the entire chromosomal DNA might be associated to MVs.

## Results

**Figure 1:**
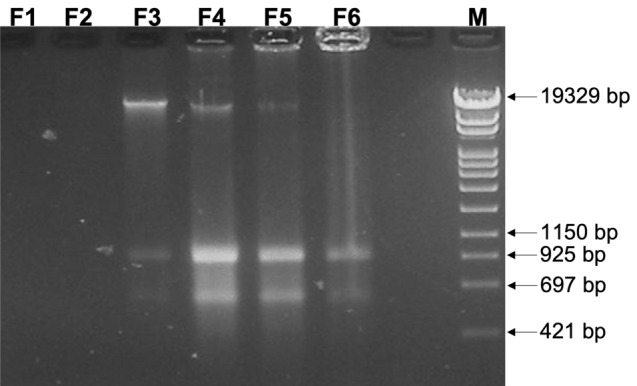
Electrophoretic analysis of the nucleic acids associated to fractions F3 and F4 of *S. coelicolor* MVs. M: DNA Molecular Weight Marker IV (Roche).

Two main populations of MVs, namely F3 and F4 MVs, were isolated and purified from liquid cultures of *S. coelicolor* according to the protocol reported in Materials and Methods. These two MV populations were previously characterized: besides having different diameters (average sizes were about 100 nm and 200 nm in F3 and F4, respectively), they differed also for the composition of their protein and metabolite cargos^4^. All six gradient fraction were directly loaded on a 1% (w/v) agarose gel and this electrophoresis analysis revealed the presence of three main bands: two smaller bands corresponding to approximately 900 bp and 500 bp, respectively, and a higher molecular weight band of approximately 19 kb (Fig. 1). According to previous works, F3 and F4 were chosen for further investigations as they are the most MV-enriched gradient fractions^4,37^. Moreover, presence of MVs in F3 and F4 was confirmed through Dynamic Light Scattering (DLS) analysis (Supplementary Fig. S1).

The relative intensities of the three bands showed an opposite pattern between the two MV populations: in F3, the 19 kb-band appeared brighter in comparison to the others, while in the F4 fraction the intensities of the 900 bp and 500 bp bands were stronger than the high molecular weight band. In order to identify the nucleic acids present in the F3 and F4 fractions, they were treated with either RNase or DNase. Data obtained revealed that the two smaller bands of both fractions corresponded to RNA, while the high-molecular-weight nucleic acid was DNA (Supplementary Fig. 2).

**Figure 2:**
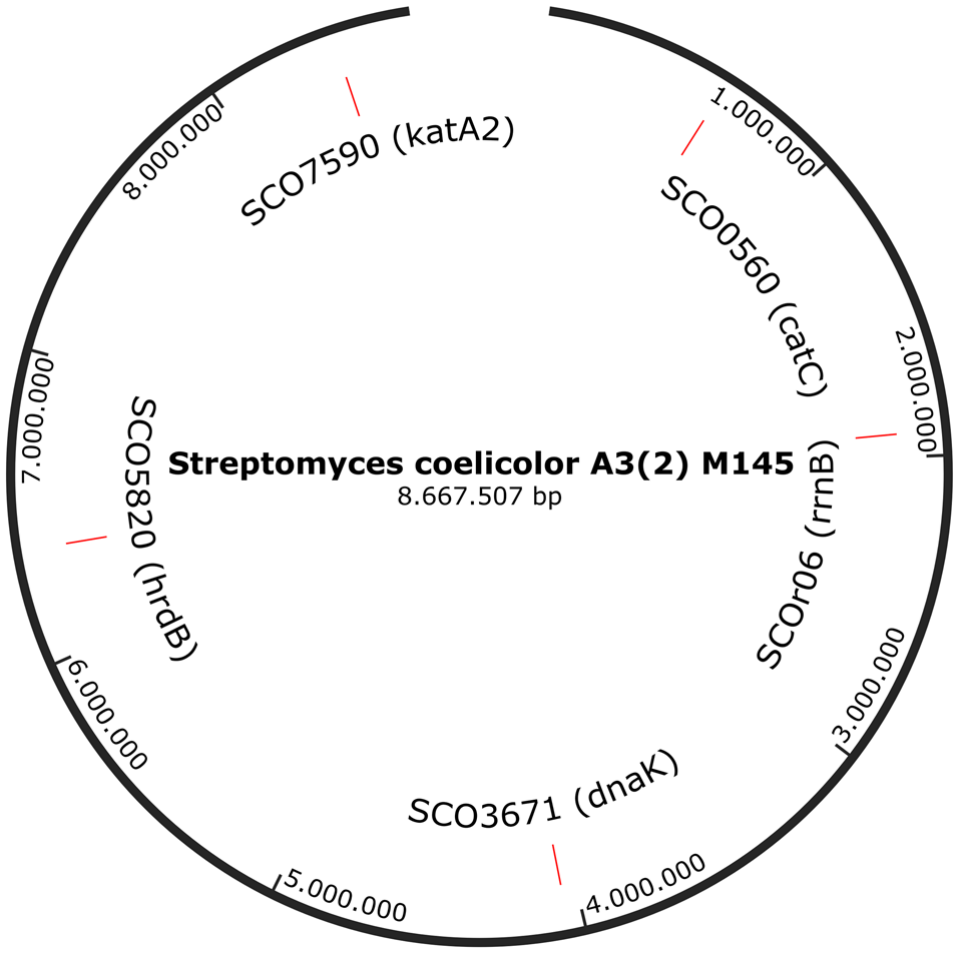
Map of *S. coelicolor* A3(2) M145 chromosome. Positions of *katA2*, *catC*, *dnaK*, *hrdB*, and *rrnB* are reported. The map was prepared with the software SnapGene Viewer using the sequence of *S. coelicolor* A3(2) available in GenBank with accession number AL645882.2.

PCR experiments aiming to identify the DNA content of F3 and F4 were performed by amplifying portions of genes mapping in different regions of *S. coelicolor* chromosome. To this purpose *katA2*, *catC*, *dnaK*, *hrdB*, and *rrnB* were chosen as target genes (Fig. 2).

Data obtained revealed that five amplicons of the expected size were obtained from both fractions suggesting that the DNA content of F3 and F4 mirrored the *S. coelicolor* chromosome (Supplementary Fig. S3).

In order to check which regions of the *S. coelicolor* chromosome were associated to the MVs, Next-Generation Sequencing (NGS) of DNA from F3 was performed: F3 was chosen for this analysis because it was the most DNA-enriched fraction. DNA sequencing resulted in 7,692,359 pair-end sequences, with 3,831,272 of them passing the trimming step. 411 contigs were obtained during the assembly step, with N50 = 62,711 bp, GC% = 72.18%, and a total length of 8,580,830 bp. Only the 255 contigs longer than 1000 bp and accounting for a total of 8,545,699 bp, were used for the further scaffolding, which was carried out using the MeDuSa software (see Materials and methods). The resulting scaffold contained a single contig of 8,558,499 bp with GC% = 72.07% and 128 gaps. The average nucleotide identity (ANI) analysis confirmed that the assembled genome belonged to *S. coelicolor*: indeed, 13 out of 15 reference genomes had an ANI value > 99.9%, as reported in Supplementary Table S2. The two lowest ANI values were probably due to the quality of the corresponding reference genomes; indeed, they had larger numbers of contigs and smaller N50 values (Supplementary Tables S1–S2).

**Figure 3:**
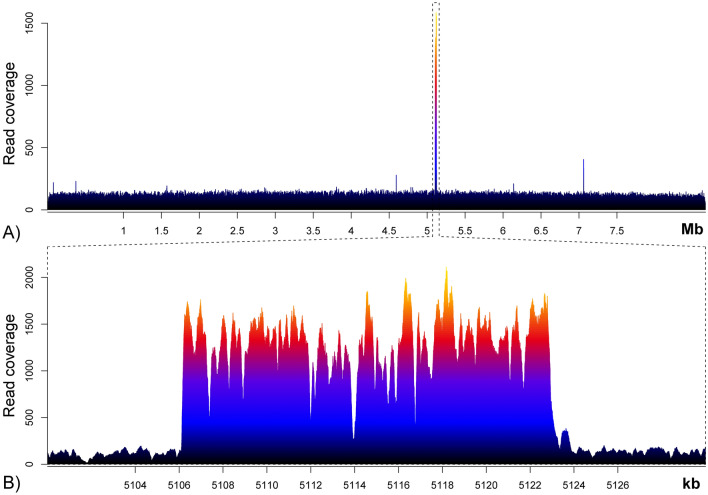
Mapping of sequencing reads on the reference chromosome sequence of *S. coelicolor* A3(2). (**A**) Sequence coverage of *S. coelicolor* A3(2) chromosome. (**B**) Zoom of the region comprised between positions 5,100,000 and 5,130,000 of the reference genome AL645882.2.

When sequencing reads were mapped on the reference chromosome sequence of *S. coelicolor* A3(2) (Fig. 3), the analysis showed that the reads covered the whole genome sequence, as suggested by PCR data.

It is noteworthy that, while the average read coverage was 117.5X, a ~ 17 kb-long region of the chromosome (from position 5,106,161 to 5,122,930 of the reference genome with accession number AL645882.2) was overrepresented, having a local average coverage of 1288.5X and including the 25 ORFs listed in Table 1.

**Table 1:** List of the ORFs mapping in the overrepresented region of *S. coelicolor* chromosome. Products were inferred from UniProtKB.

ORF	Product
SCO4674	Glyoxalase_6 domain-containing protein
SCO4675	Uncharacterized protein
SCO4676	Uncharacterized protein
SCO4677	Putative regulatory protein
SCO4678	HTH cro/C1-type domain-containing protein
SCO4679	DUF397 domain-containing protein
SCO4680	Putative DNA-binding protein
SCO4681	Putative short chain dehydrogenase
SCO4682	Putative tautomerase
SCO4683	Glutamate dehydrogenase
SCO4684	Cold shock protein
SCO4685	Putative DEAD-box RNA helicase
SCO4686	Uncharacterized protein
SCO4687	Uncharacterized protein
SCO4688	HTH merR-type domain-containing protein
SCO4689	Uncharacterized protein
SCO4690	Putative membrane protein
SCO4691	Putative membrane protein
SCO4692	Uncharacterized protein
SCO4693	Putative membrane protein
SCO4694	Uncharacterized protein
SCO4695	PPM-type phosphatase domain-containing protein
SCO4696	Uncharacterized protein
SCO4697	Putative integral membrane protein
SCO4698	Putative insertion element IS1652 transposase

Overall, these data demonstrated that the DNA content of the MV population is representative of the entire *S. coelicolor* chromosome.

## Discussion

The aim of this work was to sequence and to analyze the DNA cargo carried by the MVs of *S. coelicolor* as we have demonstrated for the first time that two main populations of MVs isolated from liquid cultures of *S. coelicolor* contain nucleic acids. In particular, one of them (i.e., fraction F3) is enriched with DNA and the other one (i.e., fraction F4) with RNA. Moreover, DNA sequencing demonstrated that the DNA carried by the MVs of fraction F3 is representative of the whole *S. coelicolor*. This result is in agreement with previous works on outer membrane vesicles (OMVs) of *Pseudomonas aeruginosa* and *Dinoroseobacter shibae*, showing the presence of the whole genome in OMVs^15,38^: in addition, it was shown that in *D. shibae* a specific DNA region of its chromosome was overrepresented, reminding the pattern observed here in *S. coelicolor*^38^; however, while in the case of *D. shibae* that region included the terminus of replication (*ter*) of its circular chromosome and the overrepresentation could be due to repair of overreplicated DNA, it is still not clear whether this sequence enrichment has a biological meaning in the case of *S. coelicolor*. For example, this region of *S. coelicolor* chromosome does not include neither the origin of replication (*oriC*), whose position is 4,269,853–4,272,747 in the reference sequence^39^, nor the known ParB-binding sites (i.e., *parS*) which map in a ~ 400 kb-spanning region centered on the *oriC*^40^.

Altogether, these findings strengthen the concept that production of (O)MVs might represent a widespread HGT mechanism, encompassing both Gram-negative and Gram-positive bacteria, and contributing to the evolution of genes and genomes. *Streptomyces* genus is among the main producers of bioactive compounds, including antibiotics used to treat human infections; thus, presence of chromosomal DNA in *S. coelicolor* MVs is of relevant interest. Indeed, antibiotic resistance genes (ARGs) are usually part of biosynthetic gene clusters and can be spread in the environment and be acquired, either directly or indirectly, by other bacteria, including pathogens. At this regard, Jiang et al.^41^ have suggested the possible HGT of ARGs from *Streptomyces* to Proteobacteria, including pathogens^41^.

Different studies suggested that DNA can be localized in the lumen and/or on the surface of MVs: in both cases, independently of its localization, DNA seems to be able to mediate HGT^9,15,42^. Even in case of DNA associated to the surface of (O)MVs, it was demonstrated that this DNA is involved in HGT: for instance, it was reported that DNase treatment of OMVs from *Porphyromonas gingivalis* impairs the efficiency of gene transfer^8,43^. Localization of DNA associated to MVs probably depends on their biogenesis, regarding either viable cells or dying ones: for example, in the latter case, DNA could also be associated to MVs by sticking on the surface of released vesicles^44^. This kind of cargo loading might be likely in *Streptomyces*, as programmed cell death is known and exerts a relevant role in the morpho-physiological differentiation of these bacteria^45,46^.

Three possible mechanisms of MV-mediated HGT were proposed: (i) MV moves closer to the target cell and the DNA on its surface is acquired by the cell by natural competence; (ii) MV associated with the membrane of the target cell undergoes lysis and DNA is acquired by natural competence; (iii) the MV membrane fuses with that of the target cell and the cargo is thus delivered into the cell^42,47^.

The role of MV DNA in genome evolution is tempting and promising; however, as DNA can be localized on the external surface of MVs, it cannot a priori be excluded that DNA might primarily exert an architectural function in MV formation and/or providing the mechanical support for shape maintenance once MVs are secreted. Moreover, *Streptomyces* MV DNA could have a possible role to promote mycelial cell adhesion to organic and inorganic substrates or in mycelial cell–cell interaction determining clamp and pellet formation in liquid cultures^48^. It should not surprise that DNA in MVs can exert other functions besides HGT: indeed, it is known that, in human cells, DNA associated to extracellular vesicles can have multiple roles, such as induction of inflammatory and immune responses by activating pro-inflammatory signaling pathways, and maintenance of cellular homeostasis through the clearance of damaged DNA that might induce apoptosis^49^.

Besides the physiological role of DNA associated to MVs, new transformation methods and biotechnological applications could take advantage of DNA packaging and delivery through MVs, especially in the case of bacteria being recalcitrant to the more diffuse genetic engineering methods. To this purpose, future works should identify the molecular mechanisms taking part in DNA selection during production of MVs.

We are completely aware that several questions remain opened. Indeed, it should be clarified, for example, if each MV contains (at least) an entire copy of the chromosome, if it is fragmented or not, and if it is bound and packed by proteins. In spite of this, in our opinion, this work represents an important step forward for the comprehension of the mechanisms responsible for the evolution of bacterial species, especially for those species whose genome is complex, such as the *Streptomyces* one. It is quite possible that the DNA embedded in *S. coelicolor* MVs might be sufficiently long to contain gene clusters responsible for a single or multiple metabolic pathways. If this is so, the genomes of *Streptomyces* and/or closer species might undergo fast evolutionary changes by acquiring long DNA stretches and rendering the metabolic versatility of bacteria belonging to this genus particularly consistent.

## Materials and methods

### Isolation and purification of streptomyces coelicolor MVs

The strain used in this study was *Streptomyces coelicolor* A3(2) M145 (genotype SCP1^−^ SCP2^−^)^50^*.* 10^8^ spores were inoculated in 30 mL of J medium^50^, for seed culture, and incubated for 30 h at 30 °C under shaking (200 rpm). The collected biomass was washed twice with sterile water and resuspended in 30 mL of sterile water. Then, 10 mL of the bacterial suspension were inoculated in 500 mL of minimal medium [NaNO_3_ (1 g/L), MgSO_4_·7H_2_O (0.5 g/L), KCl (0.5 g/L), KH_2_PO_4_ (1 g/L), glucose (10 g/L), trace element solution (1 mL/L), pH 7; trace element solution contained FeSO_4_·7H_2_O (1 g/100 mL), ZnCl_2_ (1 g/100 mL), and biotin (0.1 g/100 mL)] (MM)^51^, in a 2 L-baffled flask, and incubated at 30 °C for 136 h, under shaking at 180 rpm. *S. coelicolor* membrane vesicles were isolated and purified as described previously^4^. In brief, the culture supernatant was filtered and subsequently concentrated using an Amicon Ultrafiltration system with a 100-kDa exclusion filter (Millipore). Then, it was subjected to sequential centrifugations at 4000 and 15,000 g for 15 min at 4 °C to remove aggregates. The remaining supernatant was ultracentrifuged at 100,000 g (SW 40 Ti Rotor, Beckman Coulter) for 1 h at 4 °C. The pellet was suspended in sterile phosphate-buffered saline (Dulbecco’s phosphate-buffered saline—DPBS—without Ca^2+^ and Mg^2+^) and mixed with the OptiPrep solution (Sigma-Aldrich) to obtain a final 35% v/v OptiPrep solution. A six-layer (35, 30, 25, 20, 15, and 10% v/v) OptiPrep density gradient was set up in a 14 mL ultracentrifugation tube and was ultracentrifuged at 140,000 g for 16 h at 4 °C (SW 40 Ti Rotor, Beckman Coulter). The six fractions of the density gradient (F1 through F6) were collected and diluted with sterile DPBS and ultracentrifuged at 38,400 g (SW 55 Ti Rotor, Beckman Coulter) for 2 h at 4 °C. Pellets were suspended in 0.2 mL of sterile DPBS. 10 μL of all six fraction were visualized on a 1% (w/v) agarose gel. F3 and F4 were used for subsequent procedures, as they contained MVs as demonstrated previously^4^. DLS analysis was performed to confirm presence of MVs before proceeding with experiments.

### DNase- and RNase-treatment of MV fractions

5 μL of F3 and F4 MVs were treated with 1 U of DNase I recombinant, RNase-free (Roche), incubating samples at 37 °C for 30 min, in presence of the provided reaction buffer and in a final volume of 10 μL. The treatment with RNase was performed using 5 μL of F3 and F4 MVs and Rnase A (Roche), used at the final concentration of 10 ng/mL in 10 μL; in this case incubation was performed at 37 °C for 1 h. Treated and untreated samples were compared by loading the same amount of MVs on a 1% (w/v) agarose gel (10 μL of treated samples and 5 μL of untreated samples, respectively).

10 μl-aliquots of F3 and F4 MVs were treated with either DNase I recombinant, Rnase-free (Roche) or Rnase A (Roche) following the manufacturer’s instructions. Upon treatments, samples were analyzed by 1% (w/v) agarose gel electrophoresis in 1X Tris–Acetate-EDTA buffer (TAE). MassRuler DNA Ladder Mix, ready to use (Thermo Scientific) was used as DNA ladder.

### PCR analysis

SCO7590 (*katA2*), SCO0560 (*catC*), SCO3671(*dnaK*), SCO5820 (*hrdB*), and SCOr06 (*rrnB*) genes were amplified from MVs samples using the primer sets listed in Table 2. The reaction mixtures consisted of 1X PCR buffer, 1.5 mM MgCl_2_, 0.5 μM of each primer, 0.2 mM dNTPs, 1 U of Taq DNA Polymerase Recombinant (Thermo Scientific), and 1 μL of F3 or F4, used as template. For the amplification of SCO7590, SCO0560, SCO3671, and SCO5820, touchdown PCR conditions were applied as follows: initial denaturation for 3 min at 95 °C, followed by a cycle of 45 s at 95 °C, 30 s at 66 °C and 45 s at 72 °C; a cycle of 45 s at 95 °C, 30 s at 64 °C and 45 s at 72 °C; a cycle of 45 s at 95 °C, 30 s at 62 °C and 45 s at 72 °C; 30 cycles of 45 s at 95 °C, 30 s at 60 °C and 45 s at 72 °C. A final elongation step at 72 °C for 10 min terminated the program. For SCOr06 thermal cycling conditions were 94 °C for 3 min, followed by 30 cycles of 94 °C for 45 s, 50 °C for 1 min and 72 °C for 90 s, and finally 72 °C for 10 min.

**Table 2:** List of primers used in this study.

Target gene	Forward primer (5′ > 3′)	Reverse primer (5′ > 3′)	Amplicon expected length (bp)
SCO7590 (*katA2*)	AGGACCCGATGAAGTTCCAG	CATGTAGGTGTGGGAGGTGT	197
SCO0560 (*catC*)	AAGTACGGCCAGAACCTCTC	CCGGGTTGACGTAGATCAGA	241
SCO3671 (*dnaK*)	CAAGAAGCTCGGGATGTTCG	GACGGTCATCTTCTGCTCCT	151
SCO5820 (*hrdB*)	CGTCGAGGGTCTTCGGCT	CGCGAGCCCATCTCGCTG	220
SCOr06 (*rrnB*)	TACGGYTACCTTGTTACGACTT	AGAGTTTGATCMTGGCTCAG	1491

### Assembly of MV DNA sequence

DNA from F3 was sequenced by Genomix4life s.r.l. (Salerno, Italy) using the Illumina NextSeq550 platform with a 2 × 150 bp paired-end sequencing. Indexed libraries were prepared with a Nextera DNA Flex Kit (Illumina Inc.). Reads were trimmed using Trimmomatic (v. 0.36.4)^52^: adapter were removed using the ILLUMINACLIP option, then the trimming was performed in two steps using the SLIDINGWINDOW and the MINLEN (set to 90) options. Trimmed reads were assembled using SPAdes (v. 3.12.0)^53^ with the ‘—careful’ and the ‘—cov-cutoff’ (set to ‘auto’) options. Contigs with a length of > 1000 bp were scaffolded with MeDuSa^54^ using the reference genomes of *Streptomyces coelicolor* reported in Supplemetary Table S1. FastANI (v. 1.3)^55^ was used to calculate the average nucleotide identity (ANI) of orthologous gene pairs in the scaffold and reference genomes.

Trimmed reads were finally mapped on the reference chromosome sequence of *S. coelicolor* A3(2) (available in GenBank with accession number AL645882.2) using Bowtie2 (v. 2.4.2)^56^, and duplicate reads were removed with Samtools markdup (v. 1.13)^57^. The coverage across the entire genome was computed using the ‘genomecov’ function of BEDTools (v. 2.30.0)^58^ and data were visualized using the Sushi R package (v. 1.34.0)^59^. All these analyses were performed with software available in the Galaxy platform^60^.

## Supplementary Information

Supplementary Information.

## Data Availability

Raw sequencing reads that support the findings of this study have been deposited in the Sequence Read Archive (SRA) database with the accession number SRR19648592 (https://www.ncbi.nlm.nih.gov/sra/SRR19648592). The BioProject accession number is PRJNA849152.
