# Supplementary material for: Unravelling the DNA sequences carried by Streptomyces coelicolor membrane vesicles
Source: Sci Rep. 2022 Oct 5;12:16651. doi: 10.1038/s41598-022-21002-z (PMC9534924; doi:10.1038/s41598-022-21002-z)
Supplement: Supplementary file 1 — Supplementary Information. [file 41598_2022_21002_MOESM1_ESM.pdf]

Unravelling the DNA sequences carried by *Streptomyces coelicolor* membrane vesicles

Teresa Faddetta [1], Alberto Vassallo [2,*], Sara Del Duca [3], Giuseppe Gallo [1], Renato Fani [3], Anna Maria Puglia [1]

[1] Department of Biological, Chemical and Pharmaceutical Sciences and Technology, University of Palermo, Palermo, Italy

[2] School of Biosciences and Veterinary Medicine, University of Camerino, 62032 Camerino, Italy

[3] Department of Biology, University of Florence, 50019 Sesto Fiorentino, Italy

[*] Correspondence: alberto.vassallo@unicam.it (AV)

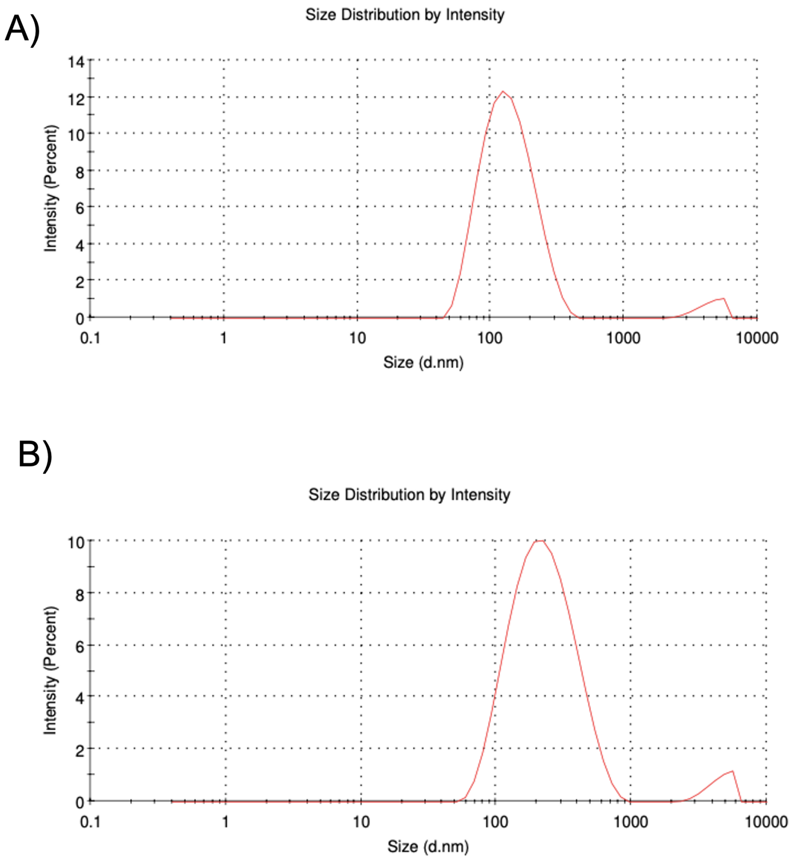

Supplementary Figure 1: Dynamic light scattering (DLS) analysis of MVs purified from *S. coelicolor*. A) F3 MVs. B) F4 MVs.

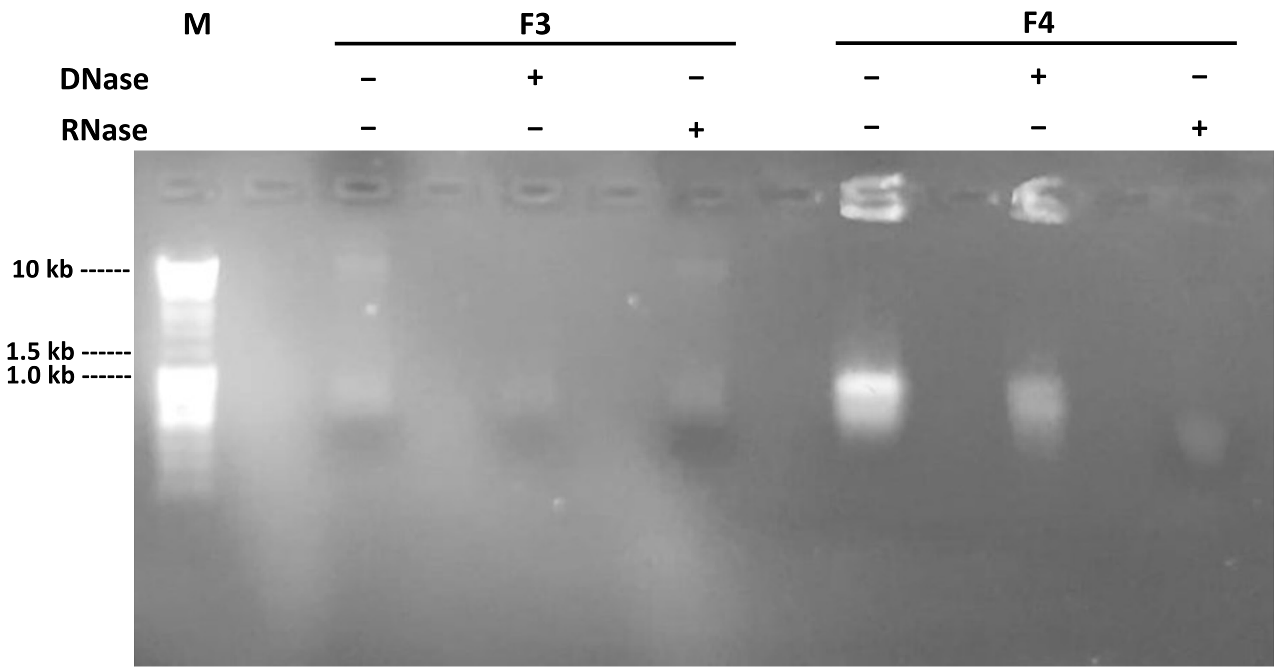

Supplementary Figure 2. Nuclease treatment of *S. coelicolor* MVs. M: MassRuler DNA Ladder Mix, ready to use (Thermo Scientific).

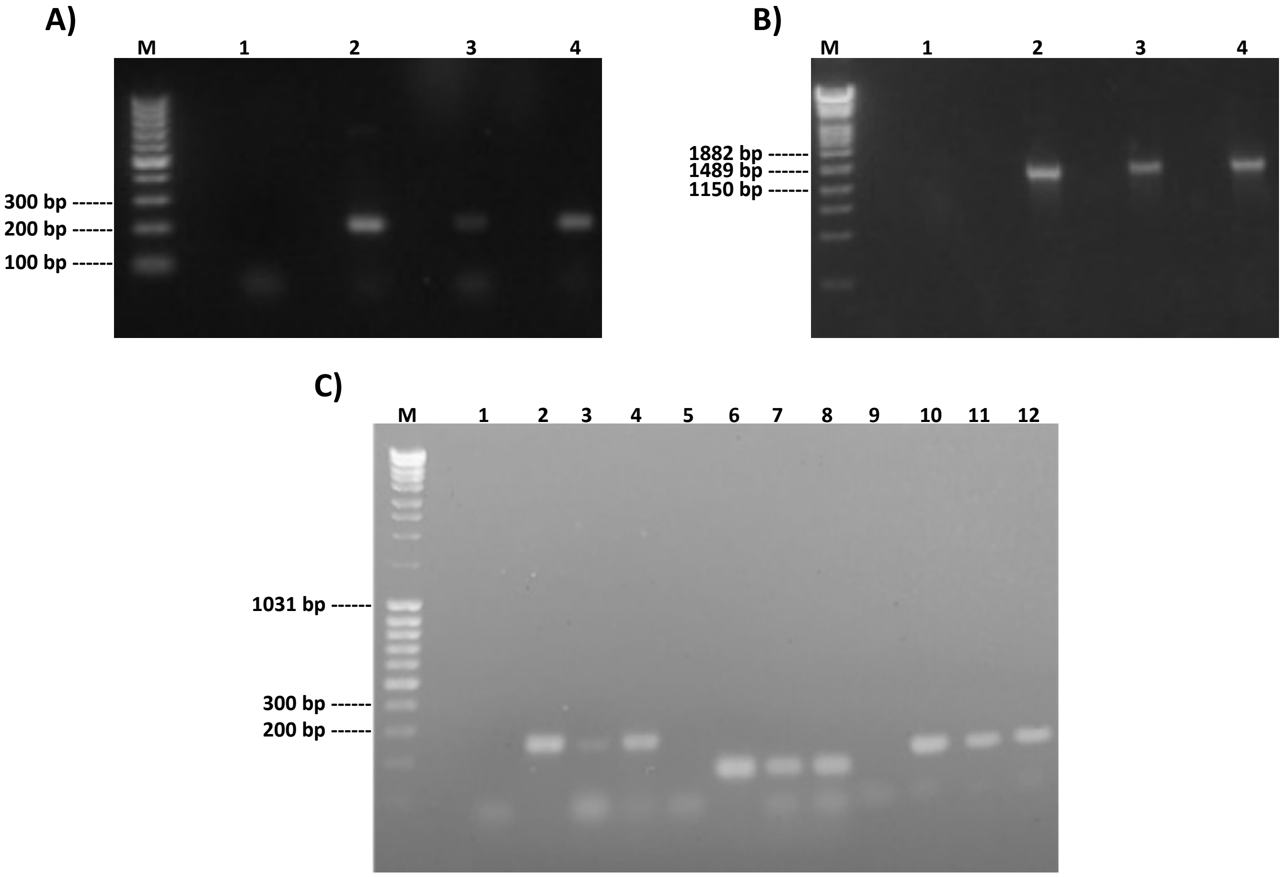

Supplementary Figure 3: PCR analysis of *S. coelicolor* MVs A) PCR analysis of *hrdB* (SCO5820). M: 100 bp DNA ladder (Invitrogen); 1: Negative control; 2: *S. coelicolor* genomic DNA; 3: F3 MVs; 4: F4 MVs. B) PCR analysis of *rrnB* (SCOr06). M: DNA Molecular Weight Marker IV (Roche); 1: Negative control; 2: *S. coelicolor* genomic DNA; 3: F3 MVs; 4: F4 MVs. C) MassRuler DNA Ladder Mix, ready to use (Thermo Scientific); 1-4 PCR analysis of *catC* (SCO0560). 1: Negative control; 2: *S. coelicolor* genomic DNA; 3: F3 MVs; 4: F4 MVs. 5-8 PCR analysis of *dnaK* (SCO3671). 5: Negative control; 6: *S. coelicolor* genomic DNA; 7: F3 MVs; 8: F4 MVs. 9-12 PCR analysis of *katA2* (SCO7590). 9: Negative control; 10: *S. coelicolor* genomic DNA; 11: F3 MVs; 12: F4 MVs.

Supplementary Table 1. Reference genomes of *S. coelicolor* used in this work.

GenBank assembly accession	Number of contigs	Length (bp)	N50 (bp)
GCA_013307045.1	87	8476974	356919.0
GCA_008124905.1	62	8585353	269139
GCA_006681775.1	2477	6708051	4850
GCA_008124965.1	66	8584648	269524.0
GCA_008124985.1	65	8584323	269524
GCA_008931305.1	1	8667664	8667664.0
GCA_000203835.1	3	9054847	8667507
GCA_004368945.1	90	8581526	230812.0
GCA_008125015.1	64	8592989	288786
GCA_008125035.1	64	8584931	266649
GCA_013317105.1	1	8585093	8585093
GCA_004368985.1	89	8581744	230600.0
GCA_008124915.1	62	8580007	288565
GCA_008124975.1	65	8584079	269524
GCA_013363625.1	334	7495065	76636

Supplementary Table 2. Output of the ANI analysis.

Reference Genome	ANI Value	Count of Bidirectional Fragment Mappings	Total Query Fragments
GCA_013307045.1	99.9597	2807	2852
GCA_008124905.1	99.9566	2807	2852
GCA_006681775.1	99.9563	2756	2852
GCA_008124965.1	99.956	2811	2852
GCA_008124985.1	99.9559	2806	2852
GCA_008931305.1	99.9555	2808	2852
GCA_000203835.1	99.955	2795	2852
GCA_004368945.1	99.9542	2790	2852
GCA_008125015.1	99.952	2805	2852
GCA_008125035.1	99.9517	2812	2852
GCA_013317105.1	99.947	2837	2852
GCA_004368985.1	99.9443	2838	2852
GCA_008124915.1	99.9402	2782	2852
GCA_008124975.1	85.4288	929	2852
GCA_013363625.1	84.7235	1639	2852
